# Crystal structure of 5-[4-(di­methyl­amino)­phen­yl]-3-(4-methyl­phen­yl)-4,5-di­hydro-1*H*-pyrazole-1-carbaldehyde

**DOI:** 10.1107/S2056989015023294

**Published:** 2015-12-09

**Authors:** Farook Adam, Seranthimata Samshuddin, Nadiah Ameram, Subramaya, Laxminarayana Samartha

**Affiliations:** a School of Chemical Sciences, Universiti Sains Malaysia, 11800 Pulau Pinang, Malaysia; b Department of PG Studies in Chemistry, Alva’s College, Moodbidri, Karnataka 574 227, India

**Keywords:** crystal structure, substituted pyrazole, pyrazole derivatives, pharmacological properties

## Abstract

The title compound, C_19_H_21_N_3_O, comprises a central pyrazole ring which is N-connected to an aldehyde group and C-connected twice to substituted benzene rings. The pyrazole ring is twisted on the C—C single bond, and the least-squares plane through this ring forms dihedral angles of 82.44 (5) and 4.52 (5)° with the (di­methyl­amino)­benzene and *p*-tolyl rings, respectively. In the crystal, weak C—H⋯O hydrogen bonds link mol­ecules into supra­molecular tubes along the *b* axis.

## Related literature   

For pharmacological properties of pyrazole derivatives, see: Sarojini *et al.* (2010 ▸); Samshuddin *et al.* (2012 ▸). For their industrial applications, see: Wiley *et al.* (1958 ▸); Lu *et al.* (1999 ▸). For related structures, see Fun *et al.* (2010 ▸); Baktır *et al.* (2011 ▸).
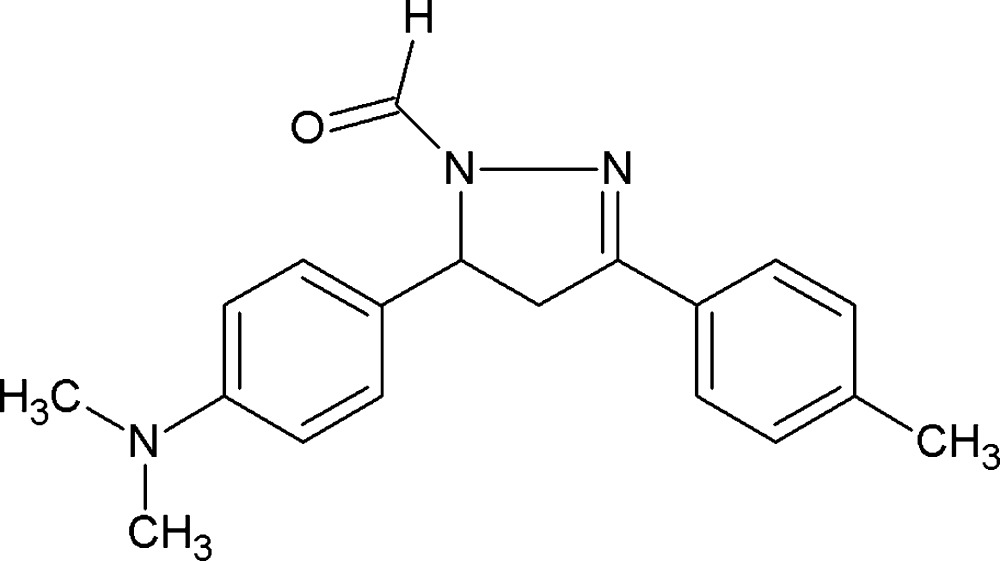


## Experimental   

### Crystal data  

C_19_H_21_N_3_O
*M* *_r_* = 307.39
Monoclinic, 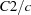

*a* = 21.9524 (15) Å
*b* = 6.2511 (4) Å
*c* = 24.1521 (16) Å
β = 106.3069 (9)°
*V* = 3181.0 (4) Å^3^
*Z* = 8
Mo *K*α radiation
μ = 0.08 mm^−1^
*T* = 100 K
0.45 × 0.26 × 0.15 mm

### Data collection  

Bruker APEX DUO CCD area-detector diffractometer
Absorption correction: multi-scan (*SADABS*; Sheldrick, 1996 ▸) *T*_min_ = 0.921, *T*_max_ = 0.962
27492 measured reflections
4750 independent reflections
4090 reflections with *I* > 2σ(*I*)
*R*_int_ = 0.028

### Refinement  

*R*[*F* ^2^ > 2σ(*F* ^2^)] = 0.043
*wR*(*F* ^2^) = 0.122
*S* = 1.04
4750 reflections
211 parameters
H-atom parameters constrained
Δρ_max_ = 0.37 e Å^−3^
Δρ_min_ = −0.22 e Å^−3^

### 

Data collection: *APEX2* (Bruker, 2014 ▸); cell refinement: *SAINT* (Bruker, 2014 ▸); data reduction: *SAINT*; program(s) used to solve structure: *SHELXS2013* (Sheldrick, 2008 ▸); program(s) used to refine structure: *SHELXL2014* (Sheldrick, 2015 ▸); molecular graphics: *SHELXTL* (Sheldrick, 2008 ▸); software used to prepare material for publication: *PLATON* (Spek, 2009 ▸).

## Supplementary Material

Crystal structure: contains datablock(s) I, New_Global_Publ_Block. DOI: 10.1107/S2056989015023294/tk5413sup1.cif

Structure factors: contains datablock(s) I. DOI: 10.1107/S2056989015023294/tk5413Isup2.hkl

Supporting information file. DOI: 10.1107/S2056989015023294/tk5413Isup3.cml

**Fig. 1.**
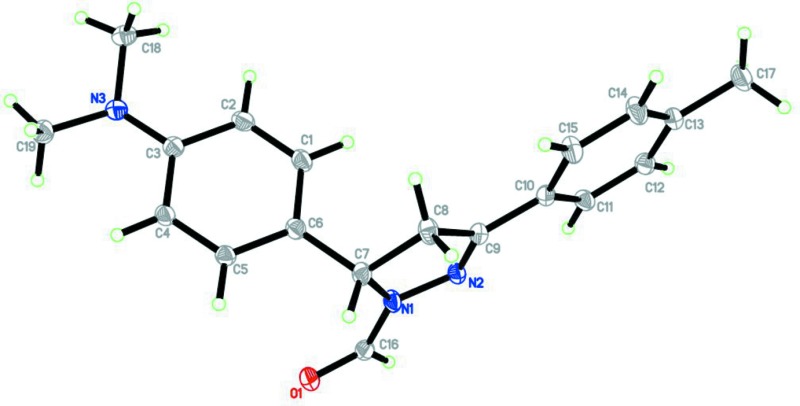
The molecular structure of (I), showing the atom labels and 50% probability displacement ellipsoids.

**Fig. 2.**
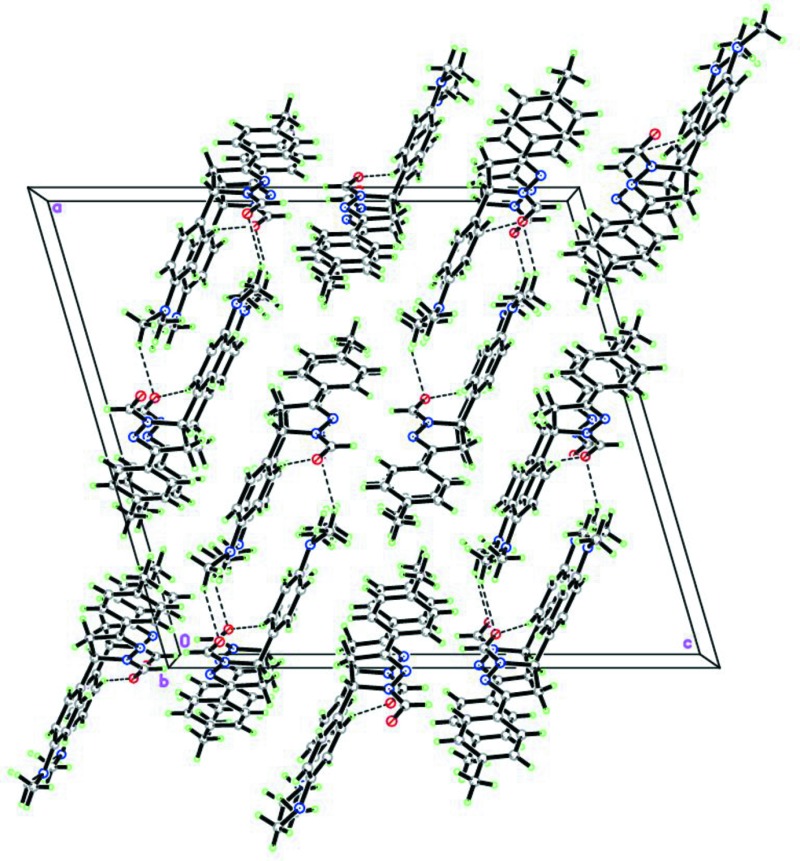
The crystal packing of (I), viewed along the b axis.

. DOI: 10.1107/S2056989015023294/tk5413fig1.tif
The mol­ecular structure of (I), showing the atom labels and 50% probability displacement ellipsoids.

b . DOI: 10.1107/S2056989015023294/tk5413fig2.tif
The crystal packing of (I), viewed along the *b* axis.

CCDC reference: 1440601

Additional supporting information:  crystallographic information; 3D view; checkCIF report

## Figures and Tables

**Table 1:** Hydrogen-bond geometry (Å, °)

*D*—H⋯*A*	*D*—H	H⋯*A*	*D*⋯*A*	*D*—H⋯*A*
C1—H1*A*⋯O1^i^	0.95	2.52	3.4175 (12)	158
C19—H19*A*⋯O1^ii^	0.98	2.45	3.3902 (15)	161

Symmetry codes: (i) 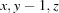; (ii) 
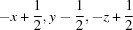
.

## supplementary crystallographic information

### S1. Introduction

Pyrazolyl derivatives are well known for their versatile pharmacological activities (Sarojini *et al.*, 2010; Samshuddin *et al.*, 2012). In addition, many 1,3,5-tri­aryl-2-pyrazolyls have a variety of industrial applications such as functioning as scintillation solutes (Wiley *et al.*, 1958) and fluorescent agents (Lu *et al.*, 1999). The crystal structures of some pyrazolyls containing a *N*-alkyl chain *viz*., 3,5-bis­(4-fluoro­phenyl)-4,5-di­hydro-1*H*-pyrazole-1-carbaldehyde (Baktır *et al.*, 2011) and 1-[3,5-bis­(4-fluoro­phenyl)-4,5-di­hydro-1*H*-pyrazol-1-yl]ethanone (Fun *et al.*, 2010) have been reported. In view of the importance of pyrazolines, the title compound (I) was prepared and its crystal structure reported.

### S2. Supra­molecular features

The asymmetric unit of (I) consists of a single crystallographic independent molecule as shown in Fig. 1. The pyrazoline ring (N1/N2/C7/C8/C9) is twisted about the C8—C7 bond [*Q*2 = 0.0964 (10) Å and φ2 = 133.5 (6)°] with maximum deviations of 0.057 (1) and −0.053 (1) Å from its mean plane for atoms C7 and C8, respectively. The methyl-substituted phenyl ring (C10–C15) and di­methyl­amino-substituted phenyl ring (C1–C6) make dihedral angles of 4.52 (5) and 82.44 (5)°, respectively, with the pyrazoline ring. In crystal, molecules are connected by weak C—H···O hydrogen bonds into one-dimensional spiral-like chains (Fig. 2), propagating along the crystallographic *b*-axis.

### S3. Synthesis and crystallization

A mixture of (2*E*)-3-[4-(di­methyl­amino)­phenyl]-1-(4-methyl­phenyl)­prop-2-en-1-one (2.65 g, 0.01 mol) and hydrazine hydrate (1 ml) in 30 ml formic acid was refluxed for 6 h. The reaction mixture was cooled and poured into 250 ml ice-cold water. The precipitate formed was collected by filtration and purified by recrystallization from ethanol. Single crystals were grown from ethyl acetate by slow evaporation (m.p 473–476 K; yield: 68%).

### S4. Refinement

The carbon-bound H-atoms were placed in calculated positions (C—H = 0.95–1.00 Å) and were included in the refinement in the riding model approximation, with *U*_iso_(H) set to 1.2–1.5*U*_equiv_(C) A rotating group model was applied to methyl groups.

### Crystal data

**Table:**

C_19_H_21_N_3_O	*F*(000) = 1312
*M**_r_* = 307.39	*D*_x_ = 1.284 Mg m^−^^3^
Monoclinic, *C*2/*c*	Mo *K*α radiation, λ = 0.71073 Å
*a* = 21.9524 (15) Å	Cell parameters from 9179 reflections
*b* = 6.2511 (4) Å	θ = 3.0–30.3°
*c* = 24.1521 (16) Å	µ = 0.08 mm^−^^1^
β = 106.3069 (9)°	*T* = 100 K
*V* = 3181.0 (4) Å^3^	Block, colourless
*Z* = 8	0.45 × 0.26 × 0.15 mm

### Data collection

**Table:**

Bruker APEX DUO CCD area-detector diffractometer	4750 independent reflections
Radiation source: fine-focus sealed tube	4090 reflections with *I* > 2σ(*I*)
Graphite monochromator	*R*_int_ = 0.028
φ and ω scans	θ_max_ = 30.3°, θ_min_ = 1.8°
Absorption correction: multi-scan (*SADABS*; Sheldrick, 1996)	*h* = −30→29
*T*_min_ = 0.921, *T*_max_ = 0.962	*k* = −8→8
27492 measured reflections	*l* = −34→34

### Refinement

**Table:**

Refinement on *F*^2^	0 restraints
Least-squares matrix: full	Hydrogen site location: inferred from neighbouring sites
*R*[*F*^2^ > 2σ(*F*^2^)] = 0.043	H-atom parameters constrained
*wR*(*F*^2^) = 0.122	*w* = 1/[σ^2^(*F*_o_^2^) + (0.0683*P*)^2^ + 1.9935*P*] where *P* = (*F*_o_^2^ + 2*F*_c_^2^)/3
*S* = 1.04	(Δ/σ)_max_ < 0.001
4750 reflections	Δρ_max_ = 0.37 e Å^−^^3^
211 parameters	Δρ_min_ = −0.22 e Å^−^^3^

### Special details

**Table:**

Geometry. All e.s.d.'s (except the e.s.d. in the dihedral angle between two l.s. planes) are estimated using the full covariance matrix. The cell e.s.d.'s are taken into account individually in the estimation of e.s.d.'s in distances, angles and torsion angles; correlations between e.s.d.'s in cell parameters are only used when they are defined by crystal symmetry. An approximate (isotropic) treatment of cell e.s.d.'s is used for estimating e.s.d.'s involving l.s. planes.

### Fractional atomic coordinates and isotropic or equivalent isotropic displacement parameters (Å^2^)

**Table:**

	*x*	*y*	*z*	*U*_iso_*/*U*_eq_	
O1	0.06265 (4)	0.29904 (12)	0.11364 (3)	0.02109 (17)	
N1	0.01154 (4)	−0.01958 (13)	0.10464 (3)	0.01627 (17)	
N2	−0.01890 (4)	−0.18157 (13)	0.06779 (3)	0.01565 (17)	
N3	0.25348 (4)	−0.20563 (14)	0.32043 (4)	0.01865 (18)	
C1	0.09931 (5)	−0.28895 (16)	0.21033 (4)	0.01817 (19)	
H1A	0.0779	−0.4002	0.1857	0.022*	
C2	0.15816 (5)	−0.33020 (16)	0.24891 (4)	0.01840 (19)	
H2A	0.1757	−0.4699	0.2508	0.022*	
C3	0.19237 (5)	−0.16856 (15)	0.28528 (4)	0.01578 (18)	
C4	0.16344 (5)	0.03445 (15)	0.28189 (4)	0.01599 (18)	
H4A	0.1847	0.1467	0.3062	0.019*	
C5	0.10411 (5)	0.07238 (15)	0.24333 (4)	0.01551 (18)	
H5A	0.0858	0.2108	0.2418	0.019*	
C6	0.07085 (5)	−0.08737 (15)	0.20693 (4)	0.01555 (18)	
C7	0.00689 (5)	−0.04206 (15)	0.16474 (4)	0.01606 (18)	
H7A	−0.0115	0.0911	0.1764	0.019*	
C8	−0.04155 (5)	−0.22678 (17)	0.15670 (4)	0.01855 (19)	
H8A	−0.0255	−0.3427	0.1850	0.022*	
H8B	−0.0828	−0.1755	0.1607	0.022*	
C9	−0.04769 (4)	−0.30150 (15)	0.09584 (4)	0.01509 (18)	
C10	−0.08377 (4)	−0.48998 (15)	0.06946 (4)	0.01530 (18)	
C11	−0.08705 (5)	−0.55364 (16)	0.01292 (4)	0.01714 (19)	
H11A	−0.0660	−0.4718	−0.0093	0.021*	
C12	−0.12078 (5)	−0.73494 (16)	−0.01069 (4)	0.01826 (19)	
H12A	−0.1226	−0.7754	−0.0490	0.022*	
C13	−0.15214 (5)	−0.85946 (16)	0.02091 (4)	0.0189 (2)	
C14	−0.14841 (5)	−0.79645 (18)	0.07700 (5)	0.0228 (2)	
H14A	−0.1690	−0.8797	0.0993	0.027*	
C15	−0.11508 (5)	−0.61392 (17)	0.10118 (4)	0.0203 (2)	
H15A	−0.1136	−0.5733	0.1394	0.024*	
C16	0.03751 (4)	0.14662 (16)	0.08404 (4)	0.01661 (18)	
H16A	0.0363	0.1457	0.0444	0.020*	
C17	−0.18897 (6)	−1.05622 (18)	−0.00455 (5)	0.0265 (2)	
H17A	−0.1673	−1.1291	−0.0295	0.040*	
H17B	−0.2317	−1.0148	−0.0273	0.040*	
H17C	−0.1919	−1.1528	0.0266	0.040*	
C18	0.27313 (5)	−0.42547 (17)	0.33548 (5)	0.0208 (2)	
H18A	0.3182	−0.4282	0.3570	0.031*	
H18B	0.2665	−0.5105	0.3002	0.031*	
H18C	0.2479	−0.4857	0.3594	0.031*	
C19	0.28414 (5)	−0.04748 (17)	0.36345 (5)	0.0205 (2)	
H19A	0.3290	−0.0845	0.3794	0.031*	
H19B	0.2634	−0.0456	0.3945	0.031*	
H19C	0.2806	0.0941	0.3454	0.031*	

### Atomic displacement parameters (Å^2^)

**Table:**

	*U*^11^	*U*^22^	*U*^33^	*U*^12^	*U*^13^	*U*^23^
O1	0.0212 (3)	0.0179 (3)	0.0216 (4)	−0.0049 (3)	0.0017 (3)	−0.0014 (3)
N1	0.0194 (4)	0.0166 (4)	0.0116 (3)	−0.0050 (3)	0.0023 (3)	−0.0016 (3)
N2	0.0171 (4)	0.0146 (4)	0.0137 (4)	−0.0027 (3)	0.0017 (3)	−0.0016 (3)
N3	0.0183 (4)	0.0157 (4)	0.0204 (4)	0.0010 (3)	0.0028 (3)	0.0008 (3)
C1	0.0234 (5)	0.0151 (4)	0.0158 (4)	−0.0033 (3)	0.0051 (3)	−0.0029 (3)
C2	0.0233 (5)	0.0137 (4)	0.0184 (4)	0.0003 (3)	0.0063 (4)	−0.0006 (3)
C3	0.0185 (4)	0.0156 (4)	0.0141 (4)	−0.0009 (3)	0.0059 (3)	0.0007 (3)
C4	0.0194 (4)	0.0144 (4)	0.0138 (4)	−0.0018 (3)	0.0041 (3)	−0.0013 (3)
C5	0.0194 (4)	0.0135 (4)	0.0135 (4)	−0.0005 (3)	0.0044 (3)	−0.0003 (3)
C6	0.0187 (4)	0.0157 (4)	0.0121 (4)	−0.0021 (3)	0.0041 (3)	−0.0007 (3)
C7	0.0182 (4)	0.0175 (4)	0.0121 (4)	−0.0028 (3)	0.0037 (3)	−0.0018 (3)
C8	0.0199 (4)	0.0220 (5)	0.0145 (4)	−0.0060 (4)	0.0060 (3)	−0.0034 (3)
C9	0.0143 (4)	0.0165 (4)	0.0138 (4)	−0.0010 (3)	0.0030 (3)	−0.0012 (3)
C10	0.0143 (4)	0.0157 (4)	0.0150 (4)	−0.0014 (3)	0.0026 (3)	−0.0002 (3)
C11	0.0198 (4)	0.0162 (4)	0.0154 (4)	−0.0026 (3)	0.0051 (3)	0.0004 (3)
C12	0.0212 (4)	0.0175 (4)	0.0148 (4)	−0.0018 (3)	0.0029 (3)	−0.0011 (3)
C13	0.0182 (4)	0.0171 (4)	0.0187 (4)	−0.0039 (3)	0.0007 (3)	0.0004 (3)
C14	0.0236 (5)	0.0257 (5)	0.0194 (5)	−0.0110 (4)	0.0063 (4)	0.0002 (4)
C15	0.0214 (5)	0.0238 (5)	0.0160 (4)	−0.0073 (4)	0.0057 (4)	−0.0021 (4)
C16	0.0160 (4)	0.0167 (4)	0.0158 (4)	−0.0017 (3)	0.0024 (3)	0.0018 (3)
C17	0.0296 (5)	0.0218 (5)	0.0240 (5)	−0.0107 (4)	0.0010 (4)	−0.0018 (4)
C18	0.0230 (5)	0.0185 (5)	0.0214 (5)	0.0047 (4)	0.0069 (4)	0.0027 (4)
C19	0.0177 (4)	0.0208 (5)	0.0213 (5)	0.0008 (4)	0.0026 (4)	−0.0018 (4)

### Geometric parameters (Å, º)

**Table:**

O1—C16	1.2256 (12)	C8—H8B	0.9900
N1—C16	1.3459 (12)	C9—C10	1.4622 (13)
N1—N2	1.3892 (11)	C10—C15	1.3982 (13)
N1—C7	1.4901 (12)	C10—C11	1.4049 (13)
N2—C9	1.2889 (12)	C11—C12	1.3858 (13)
N3—C3	1.3908 (12)	C11—H11A	0.9500
N3—C19	1.4550 (13)	C12—C13	1.3991 (14)
N3—C18	1.4556 (13)	C12—H12A	0.9500
C1—C2	1.3884 (14)	C13—C14	1.3910 (15)
C1—C6	1.3986 (14)	C13—C17	1.5051 (14)
C1—H1A	0.9500	C14—C15	1.3922 (14)
C2—C3	1.4094 (13)	C14—H14A	0.9500
C2—H2A	0.9500	C15—H15A	0.9500
C3—C4	1.4112 (13)	C16—H16A	0.9500
C4—C5	1.3923 (13)	C17—H17A	0.9800
C4—H4A	0.9500	C17—H17B	0.9800
C5—C6	1.3938 (13)	C17—H17C	0.9800
C5—H5A	0.9500	C18—H18A	0.9800
C6—C7	1.5121 (13)	C18—H18B	0.9800
C7—C8	1.5446 (14)	C18—H18C	0.9800
C7—H7A	1.0000	C19—H19A	0.9800
C8—C9	1.5118 (13)	C19—H19B	0.9800
C8—H8A	0.9900	C19—H19C	0.9800
			
C16—N1—N2	120.22 (8)	C15—C10—C11	118.47 (9)
C16—N1—C7	125.80 (8)	C15—C10—C9	119.82 (9)
N2—N1—C7	113.78 (7)	C11—C10—C9	121.70 (8)
C9—N2—N1	107.81 (8)	C12—C11—C10	120.48 (9)
C3—N3—C19	119.75 (8)	C12—C11—H11A	119.8
C3—N3—C18	118.50 (8)	C10—C11—H11A	119.8
C19—N3—C18	114.70 (8)	C11—C12—C13	121.23 (9)
C2—C1—C6	121.52 (9)	C11—C12—H12A	119.4
C2—C1—H1A	119.2	C13—C12—H12A	119.4
C6—C1—H1A	119.2	C14—C13—C12	118.05 (9)
C1—C2—C3	121.18 (9)	C14—C13—C17	120.48 (9)
C1—C2—H2A	119.4	C12—C13—C17	121.47 (9)
C3—C2—H2A	119.4	C13—C14—C15	121.39 (9)
N3—C3—C2	120.99 (9)	C13—C14—H14A	119.3
N3—C3—C4	121.77 (9)	C15—C14—H14A	119.3
C2—C3—C4	117.14 (9)	C14—C15—C10	120.38 (9)
C5—C4—C3	120.88 (9)	C14—C15—H15A	119.8
C5—C4—H4A	119.6	C10—C15—H15A	119.8
C3—C4—H4A	119.6	O1—C16—N1	123.54 (9)
C4—C5—C6	121.73 (9)	O1—C16—H16A	118.2
C4—C5—H5A	119.1	N1—C16—H16A	118.2
C6—C5—H5A	119.1	C13—C17—H17A	109.5
C5—C6—C1	117.53 (9)	C13—C17—H17B	109.5
C5—C6—C7	120.91 (8)	H17A—C17—H17B	109.5
C1—C6—C7	121.55 (8)	C13—C17—H17C	109.5
N1—C7—C6	111.69 (8)	H17A—C17—H17C	109.5
N1—C7—C8	100.43 (7)	H17B—C17—H17C	109.5
C6—C7—C8	114.89 (8)	N3—C18—H18A	109.5
N1—C7—H7A	109.8	N3—C18—H18B	109.5
C6—C7—H7A	109.8	H18A—C18—H18B	109.5
C8—C7—H7A	109.8	N3—C18—H18C	109.5
C9—C8—C7	102.92 (8)	H18A—C18—H18C	109.5
C9—C8—H8A	111.2	H18B—C18—H18C	109.5
C7—C8—H8A	111.2	N3—C19—H19A	109.5
C9—C8—H8B	111.2	N3—C19—H19B	109.5
C7—C8—H8B	111.2	H19A—C19—H19B	109.5
H8A—C8—H8B	109.1	N3—C19—H19C	109.5
N2—C9—C10	121.69 (9)	H19A—C19—H19C	109.5
N2—C9—C8	114.10 (8)	H19B—C19—H19C	109.5
C10—C9—C8	124.20 (8)		
			
C16—N1—N2—C9	−170.18 (9)	C1—C6—C7—C8	38.39 (12)
C7—N1—N2—C9	4.90 (11)	N1—C7—C8—C9	9.05 (9)
C6—C1—C2—C3	−1.62 (15)	C6—C7—C8—C9	−110.93 (9)
C19—N3—C3—C2	−171.16 (9)	N1—N2—C9—C10	−179.08 (8)
C18—N3—C3—C2	−22.08 (13)	N1—N2—C9—C8	2.03 (11)
C19—N3—C3—C4	12.60 (14)	C7—C8—C9—N2	−7.57 (11)
C18—N3—C3—C4	161.68 (9)	C7—C8—C9—C10	173.57 (9)
C1—C2—C3—N3	−174.66 (9)	N2—C9—C10—C15	−179.05 (9)
C1—C2—C3—C4	1.75 (14)	C8—C9—C10—C15	−0.28 (14)
N3—C3—C4—C5	175.25 (9)	N2—C9—C10—C11	2.17 (15)
C2—C3—C4—C5	−1.13 (14)	C8—C9—C10—C11	−179.05 (9)
C3—C4—C5—C6	0.36 (14)	C15—C10—C11—C12	0.19 (15)
C4—C5—C6—C1	−0.14 (14)	C9—C10—C11—C12	178.98 (9)
C4—C5—C6—C7	−179.11 (8)	C10—C11—C12—C13	−0.23 (15)
C2—C1—C6—C5	0.77 (14)	C11—C12—C13—C14	−0.17 (15)
C2—C1—C6—C7	179.72 (9)	C11—C12—C13—C17	179.86 (9)
C16—N1—C7—C6	−72.07 (12)	C12—C13—C14—C15	0.62 (16)
N2—N1—C7—C6	113.18 (9)	C17—C13—C14—C15	−179.41 (10)
C16—N1—C7—C8	165.67 (9)	C13—C14—C15—C10	−0.67 (17)
N2—N1—C7—C8	−9.08 (10)	C11—C10—C15—C14	0.25 (15)
C5—C6—C7—N1	103.78 (10)	C9—C10—C15—C14	−178.56 (9)
C1—C6—C7—N1	−75.15 (11)	N2—N1—C16—O1	176.82 (9)
C5—C6—C7—C8	−142.69 (9)	C7—N1—C16—O1	2.38 (16)

### Hydrogen-bond geometry (Å, º)

**Table:**

*D*—H···*A*	*D*—H	H···*A*	*D*···*A*	*D*—H···*A*
C1—H1*A*···O1^i^	0.95	2.52	3.4175 (12)	158
C19—H19*A*···O1^ii^	0.98	2.45	3.3902 (15)	161

Symmetry codes: (i) *x*, *y*−1, *z*; (ii) −*x*+1/2, *y*−1/2, −*z*+1/2.
