# Supplementary figures and images for: Crystal structure of 5-[4-(di­methyl­amino)­phen­yl]-3-(4-methyl­phen­yl)-4,5-di­hydro-1H-pyrazole-1-carbaldehyde
Source: Acta Crystallogr E Crystallogr Commun. 2015 Dec 9;71(Pt 12):o1031–2. doi: 10.1107/S2056989015023294 (PMC4719963; doi:10.1107/S2056989015023294)

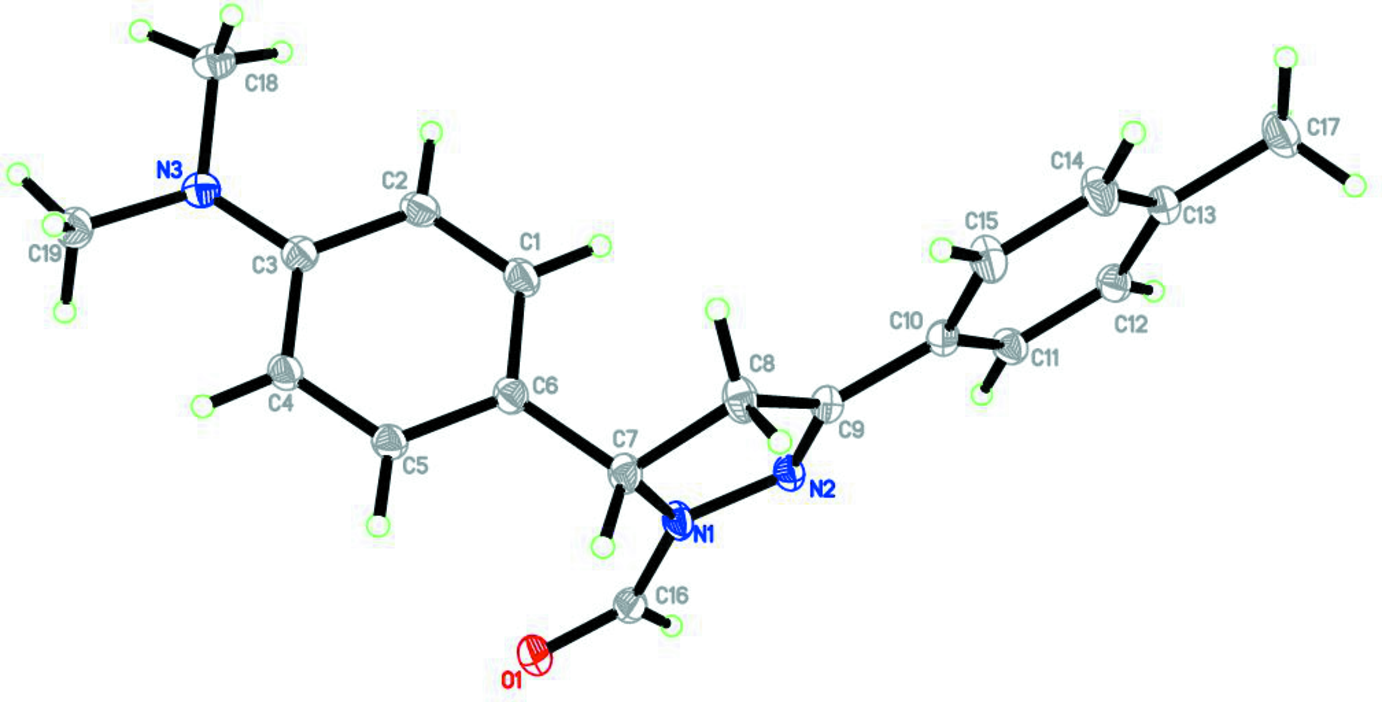

Supplement: Supplementary file 4 [file e-71-o1031-fig1.tif]

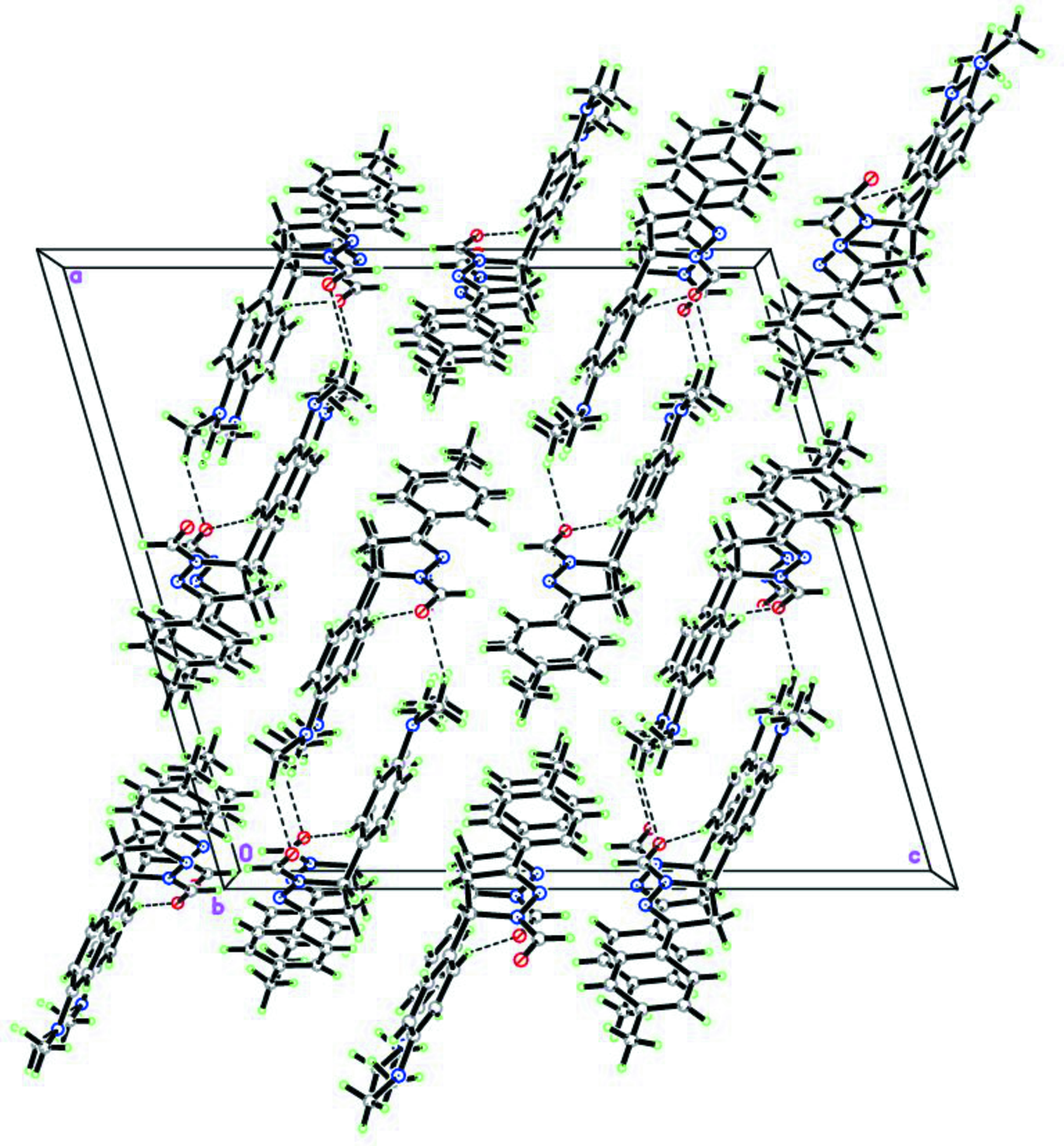

Supplement: Supplementary file 5 [file e-71-o1031-fig2.tif]
